# A model to predict unstable carotid plaques in population with high risk of stroke

**DOI:** 10.1186/s12872-020-01450-z

**Published:** 2020-04-07

**Authors:** Junxiong Yin, Chuanyong Yu, Hongxing Liu, Mingyang Du, Feng Sun, Cheng Yu, Lixia Wei, Chongjun Wang, Xiaoshan Wang

**Affiliations:** 1 Department of Neurology, Brain Hospital Affiliated to Nanjing Medical University, 264# Guangzhou road, Nanjing, 210012 Jiangsu China; 2 Department of Computer Science and Technology, Nanjing University, Nanjing, 210012 Jiangsu China

**Keywords:** High risk group of stroke individuals, Carotid unstable plaque, Risk factors, Predictive model scoring system

## Abstract

**Background:**

Several models have been developed to predict asymptomatic carotid stenosis (ACS), however these models did not pay much attention to people with lower level of stenosis (<50% or carotid plaques, especially instable carotid plaques) who might benefit from early interventions. Here, we developed a new model to predict unstable carotid plaques through systematic screening in population with high risk of stroke.

**Methods:**

Community residents who participated the China National Stroke Screening and Prevention Project (CNSSPP) were screened for their stroke risks. A total of 2841 individuals with high risk of stroke were enrolled in this study, 266 (9.4%) of them were found unstable carotid plaques. A total of 19 risk factors were included in this study. Subjects were randomly distributed into Derivation Set group or Validation Set group. According to their carotid ultrasonography records, subjects in derivation set group were further categorized into unstable plaque group or stable plaque group.

**Results:**

174 cases and 1720 cases from Derivation Set group were categorized into unstable plaque group and stable plaque group respectively. The independent risk factors for carotid unstable plaque were: male (OR 1.966, 95%CI 1.406–2.749), older age (50–59, OR 6.012, 95%CI 1.410–25.629; 60–69, OR 13.915, 95%CI 3.381–57.267;≥70, OR 31.267, 95%CI 7.472–130.83), married(OR 1.780, 95%CI 1.186–2.672), LDL-C(OR 2.015, 95%CI 1.443–2.814), and HDL-C(OR 2.130, 95%CI 1.360–3.338). A predictive scoring system was generated, ranging from 0 to 10. The cut-off value of this predictive scoring system is 6.5. The AUC value for derivation and validation set group were 0.738 and 0.737 respectively.

**Conclusions:**

For those individuals with high risk of stroke, we developed a new model which could identify those who have a higher chance to have unstable carotid plaques. When an individual’s predictive model score exceeds 6.5, the probability of having carotid unstable plaques is high, and carotid ultrasonography should be conducted accordingly. This model could be helpful in the primary prevention of stroke.

## Background

Stroke is the third leading cause of lost disability adjusted life years worldwide [1]. It is well-known that atherosclerosis is the main risk factor of cardiovascular and cerebral vascular disease [2]. Asymptomatic carotid artery stenoses are very common among residents. So, it would be helpful to screening carotid stenosis in selected population for the purpose of primary prevention of cardiovascular diseases (CVD) and stroke, although there is still controversy over it. The USPSTF recommended against screening for asymptomatic carotid stenosis (ACS) in the general adult population both in 2007 and 2014 [3, 4]. The American Society of Neuroimaging has concluded that screening for ACS is appropriate if the prevalence is greater than 20% [5].

Several studies [6, 7] demonstrated older age, smoking, high blood pressure, diabetes and others were independent risk factors for carotid stenosis (> 50% stenosis), however these models did not pay much attention to people with lower level of stenosis (<50% or carotid plaques, especially unstable plaques) who might benefit from early intervention, such as statins, antiplatelet drugs.

In order to better understand the risk factors of unstable carotid plaques, we analyzed data of 2841 individuals from China National Stroke Screening and Prevention Project (CNSSPP) Database, including carotid duplex scan, demographic information, lifestyle risk factors, medical history, family history of stroke and blood tests, etc. The purpose of this study is to identify residents with a high probability of having unstable carotid plaques, for whom primary prevention therapy might be beneficial.

## Methods

### High risk group of stroke individuals

Screening records ranging from 2012 to 2016 were provided by CNSSPP, a nationally ongoing community-based study, which was conducted by the National Project Office of Stroke Prevention and Control. Screenings were performed at 21 communities throughout the city of Nanjing.

Individuals were selected for cluster sampling. They should meet these criterions: Age ≥ 40 years old, living in the community for at least 9 month per year, and at least 85% population were included.

According to the standard established by the CNSSPP committee [8], high-risk group of stroke were defined as follows: at least 40 years old, at least three of the following risk factors, including hypertension, atrial fibrillation, smoking, dyslipidemia, diabetes mellitus, physical inactivity, overweight or obesity (BMI ≥26 kg/m^2^), and family history of stroke. Individuals who had the history of stroke or transient ischemic attack were also considered at high risk.

The definition of the eight risk factors adopts the unified standard of CNSSPP, which has been published in previous paper [8]. Hypertension was defined as a systolic blood pressure of 140 mmHg or more, diastolic blood pressure of 90 mmHg or more, self-reported hypertension diagnosed by a physician, or taking antihypertensive medications [9]. Atrial fibrillation was defined as ECG examination indicated atrial fibrillation, self-reported atrial fibrillation diagnosed by a physician, or taking anticoagulant medications. Smoking status was classified as smoking (current smoking or had a history of smoke for more than 1 year) or never smoking (never smoking or had a history of smoke for less than 1 year). Dyslipidemia was defined as: triglyceride (TG) ≥2.26 mmol/L, Total cholesterol (TC) ≥6.22 mmol/L, high-density lipoprotein cholesterol (HDL-C) < 1.04 mmol/L, low-density lipoprotein cholesterol (LDL-C) ≥4.14 mmol/L, self-reported dyslipidemia diagnosed by a physician, or taking cholesterol-lowering drugs [10]. Diabetes mellitus was defined as fasting plasma glucose ≥7.0 mmol/L, self-reported diabetes mellitus diagnosed by a physician, or taking hypoglycemic agents. Physical activity was defined as regular physical activities, lasting more than 30 min each time, more than three times a week. Overweight or obesity defined by the Working Group on Obesity in China as a body mass index (BMI) of 26 and above [11]. A family history of stroke was defined as the occurrence of stroke in parents or siblings.

Before carotid duplex scans, subjects were asked to complete a standardized CNSSPP questionnaire, including demographic information, lifestyle risk factors, medical history, and family history of stroke, which were collected through face-to-face interviews by trained staff. The questionnaire of CNSSPP is provided in Additional file 1.

### Blood test

Fast venous blood (5 mL) was collected, centrifugated at 3000 g for 10 min and stored at − 80 °C Freezer. Levels of fasting plasma glucose (FPG), homocysteine (Hcy), TC, HDL-C, LDL-C, and TG were determined using OLYMPUS AU5400 (OLYMPUS, Japan). EDTA anticoagulated whole blood samples (2 ml) were collected to determine hemoglobin A1c (HbA1c) level by TOSOH G8 (TOSOH, Japan).

### Carotid artery ultrasound screening protocol

According to Chinese stroke vascular ultrasound examination guideline [12], the duplex scan consists of ultrasound imaging of the distal common carotid artery, bulb, and proximal internal and external carotid arteries, with evaluation of a Doppler signal for 3 to 5 beats in each location on both sides. Plaque is interpreted as greater than 1.5 mm of IMT based on Doppler-derived [13]. Plaques with hypoechoic, mixed echoes, or ulceration are defined as unstable plaques. Carotid duplex examinations were performed by four experienced registered vascular technicians in Nanjing Brain Hospital, which is a Stroke Screening and Training Center. All of the vascular technicians were unaware of clinical information of subjects. Screening work was conducted in compliance with the protocol established by CNSSPP committee.

### Predictive model evaluation

The whole sample was randomly divided into a model derivation set and a model validation set, which consisted of approximately two-thirds and one-third of the sample, respectively. A comparison was performed between the two groups with t-test for continuous variables and with χ^2^ tests for categorical variables. Univariate logistic regression was carried out for each risk factor. When the value of P was less than 0.1, the variable was included in the multivariable logistic regression model (Stepwise forward). Variables with *P* values less than 0.05 are retained. According to the previous study [6], we also generated a scoring system based on the regression coefficients. The lowest coefficient in absolute value was used as denominator. The coefficient of each independent risk factor was divided by the absolute value of the lowest coefficient and then rounded up to the nearest integers. Each subject would have a score according to the model and then scores of all the subjects were used to plot receiver operator characteristic (ROC) curve, and to determine the prediction power of unstable carotid plaque, and the best cutoff score by Youden index.

We used validation set to evaluate the ability of the predictive model to discriminate between subjects with and without unstable carotid plaque, assessed using a ROC curve. ROC curve is drawn with true positive rate (sensitivity) as ordinate and false positive rate (1-specificity) as abscissa. The area under the curve (AUC) can evaluate the discrimination ability of specific screening methods. AUC values range from 0.5 to 1.0, with larger AUCs representing better performance.

After checking the normality of all continuous variables, continuous variables were presented as means (Standard deviation, SD), and categorical variables were presented as percentages. According to empirical formula of sample estimation based on multi factor analysis, more than 228 cases were needed in this study. All statistical analyses were performed using the SPSS version 20.0 software for Windows (SPSS, Inc., Chicago, IL, USA). In all statistical analyses, a *P* value < 0.05 was considered statistically significant.

## Results

**Fig. 1:**
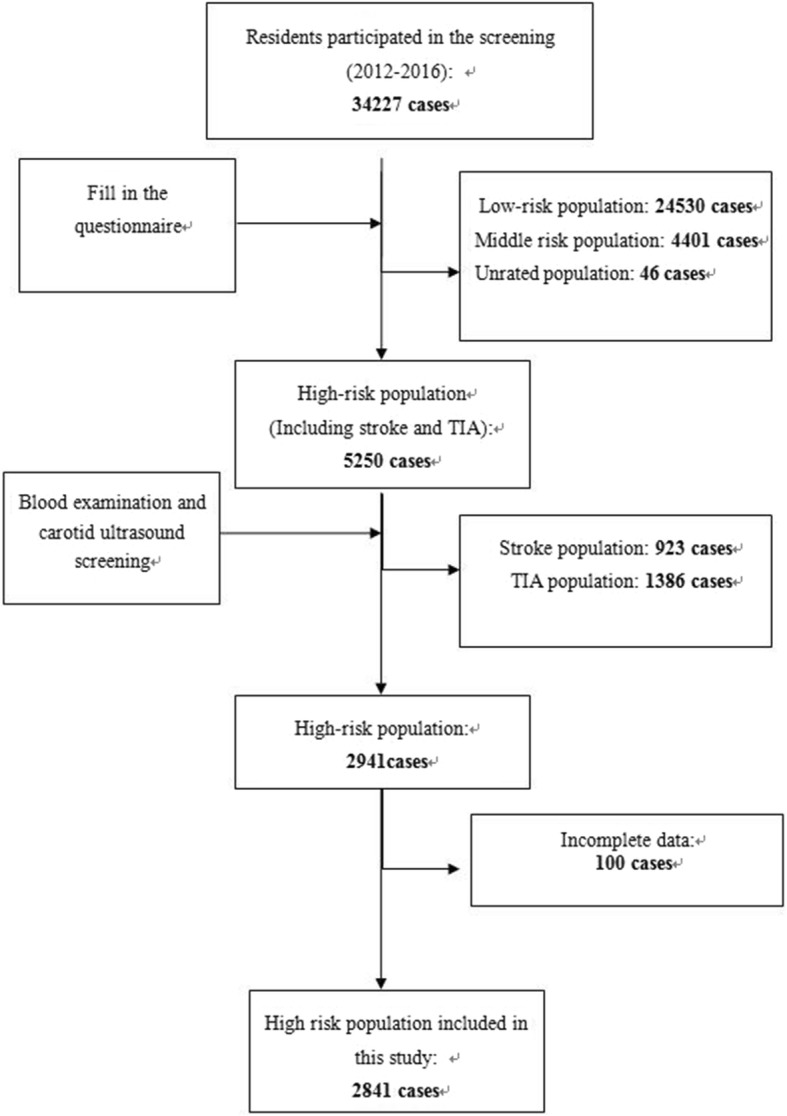
Flowchart illustrating the inclusion/exclusion of individuals in the study

During 2012–2016, 34,227 residents were enrolled in CNSSPP. According to the risk factor screening, 5250 residents were at high risk, and were screened carotid arteries by ultrasonography. A total of 2309 residents were excluded because of previous stroke or transient ischemic attack, 100 residents were excluded because of incomplete data, as shown in Fig. 1. Of the total 2841 subjects included in this study, the prevalence of carotid plaque was 35.2% (*n* = 1000), the prevalence of unstable carotid plaque was 9.4% (*n* = 266). All the 2841 subjects were randomly distributed into the derivation set group (*n* = 1894) and the validation set group (*n* = 947). Characteristics of these subjects are provided in Table 1.

**Table 1:** Demographic and Risk Factor Profiles in the Derivation and Validation Sets

Variable	Derivation Set (*n* = 1894)	Validation Set (*n* = 947)	*P*
Sex (male)	874 (46.1%)	451 (47.6%)	0.457
Mean age (years)	60.7 ± 8.6	60.4 ± 8.7	0.321
Education level (Primary school or below)	730 (38.5%)	402 (42.4%)	**0.045**
Marriage (married)	1330 (70.2%)	699 (73.8%)	0.102
Atrial fibrillation	130 (6.9%)	57 (6.0%)	0.392
Diabetes mellitus	457 (24.1%)	251 (26.5%)	0.168
Hypertension	1503 (79.4%)	743 (78.5%)	0.579
Hypercholesterolemia	741 (39.1%)	349 (36.9%)	0.241
Overweight or obesity	847 (44.7%)	408 (43.1%)	0.408
Smoking	634 (33.5%)	339 (35.8%)	0.219
Lack of Physical activity	1443 (76.2%)	727 (76.8%)	0.731
Family history of strok	602 (31.8%)	282 (29.8%)	0.276
FPG (mmol/L)	6.1 ± 1.8	6.2 ± 2.0	0.079
HbA1c (%)	6.6 ± 2.5	6.6 ± 1.9	0.872
Hcy (mmol/L)	12.2 ± 7.5	12.0 ± 6.6	0.611
TC (mmol/L)	5.1 ± 1.0	5.1 ± 1.0	0.386
LDL-C (mmol/L)	3.0 ± 0.8	3.0 ± 0.8	0.944
HDL-C (mmol/L)	1.6 ± 0.8	1.5 ± 0.7	0.600
TG (mmol/L)	1.7 ± 1.3	1.7 ± 1.4	0.699
Carotid plaque	660 (34.8%)	340 (35.9%)	0.579
Carotid instability plaque	174 (9.2%)	92 (9.7%)	0.649

The derivation set included 1894 subjects (mean age = 60.7 ± 8.6 years), 174 (9.2%) of whom had unstable carotid plaques. The clinical characteristics of this set are provided in Table 2. Men were more likely to have unstable carotid plaques. A higher proportion of subjects older than 60 had unstable carotid plaques. Subjects who were married or had a history of diabetes mellitus would have more chance to have unstable carotid plaques. Subjects with unstable carotid plaques were more likely to have a higher level of FPG, Hcy, TC, LDL-C, a lower level of HDL-C, and overweight or obesity. No significant differences were found between groups with regard to education level, atrial fibrillation, hypertension, hypercholesterolemia, smoking, and lack of physical activity, family history of stroke, HbA1c, or TG. In order to correct the influencing factors of gender and age, we established a gender and age matched control group (1:1). The results are consistent before and after correction, which are provided in Additional file 2.

**Table 2:** Comparison of Demographic and Risk Factor Profiles in Participants with and Without Carotid Instability Plaque in the Derivation Set

Variable	With Carotid Vulnerable Plaque (*n* = 174)	Without Carotid Vulnerable Plaque (*n* = 1720)	*P* Value	OR(95%CI)
Sex (male)	109 (62.6%)	765 (44.5%)	**<0.001**	**2.093 (1.518–2.887)**
Mean age (years)				
40–49	14 (8.0%)	207 (12.0%)	**<0.001**	1.00(Reference)
50–59	35 (20.1%)	512 (29.8%)	**0.018**	5.686 (1.340–24.117)
60–69	87 (50.0%)	765 (44.5%)	**<0.001**	12.933 (3.160–52.934)
≥ 70	38 (21.8%)	236 (13.7%)	**<0.001**	27.500 (6.626–114.142)
Education level (Primary school or below)	67 (38.5%)	663 (38.5%)	0.992	0.998 (0.725–1.375)
Marriage (married)	141 (81.0%)	1189 (69.1%)	**0.001**	**1.908 (1.289–2.826)**
Atrial fibrillation	13 (7.5%)	117 (6.8%)	0.740	1.106 (0.610–2.007)
Diabetes mellitus	54 (31.0%)	403 (23.4%)	**0.026**	**1.471 (1.047–2.066)**
Hypertension	145 (83.3%)	1358 (79.0%)	0.175	1.333 (0.880–2.019)
Hypercholesterolemia	67 (38.5%)	674 (39.2%)	0.861	0.972 (0.705–1.339)
Overweight or obesity	61 (35.1%)	786 (45.7%)	**0.008**	**0.641 (0.463–0.888)**
Smoking	68 (39.1%)	566 (32.9%)	0.101	1.308 (0.949–1.802)
Lack of Physical activity	134 (77.0%)	1309 (76.1%)	0.789	1.052 (0.726–1.523)
Family history of strok	54 (31.0%)	548 (31.9%)	0.824	0.962 (0.687–1.348)
FPG (mmol/L)				
≤ 6.1	101 (58.0%)	1192 (69.3%)	**0.007**	1.0(Reference)
6.11~6.99	29 (16.7%)	235 (13.7%)	**0.091**	1.456 (0.942–2.252)
≥ 7.0	44 (25.3%)	293 (17.0%)	**0.003**	1.772 (1.216–2.582)
HbA1c (> 6.5%)	14 (8.0%)	125 (7.3%)	0.959	1.109 (0.496–2.094)
Hcy(> 15 mmol/L)	30 (17.2%)	212 (12.3%)	0.122	1.393 (0.915–2.120)
TC(> 5.2 mmol/L)	91 (52.3%)	716 (41.6%)	**0.007**	**1.537 (1.125–2.101)**
LDL-C(> 3.12 mmol/L)	97 (55.7%)	704 (40.9%)	**<0.001**	**1.467 (1.223–1.761)**
HDL-C (< 1.04 mmol/L)	32 (18.4%)	194 (11.3%)	**0.006**	**1.773 (1.175–2.675)**
TG(> 1.7 mmol/L)	59 (33.9%)	574 (33.4%)	0.886	1.024 (0.737–1.424)

**Fig. 2:**
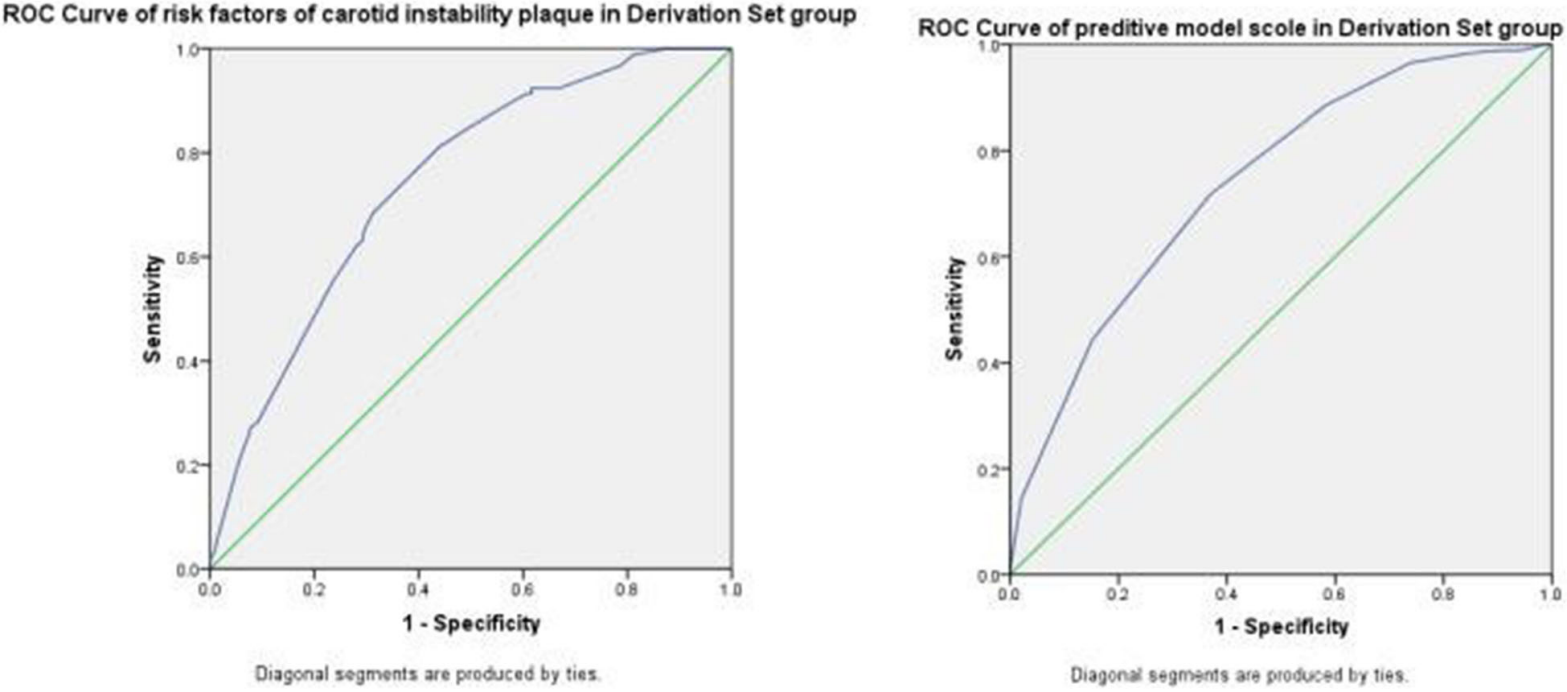
The ROC curve of Derivation Set group for risk factors and predictive model Scoring in high risk group of strokes

Table 3 shows the results of multivariable logistic regression analysis. Five variables were significantly associated with unstable carotid plaque: male (OR 1.966, 95%CI 1.406–2.749), older age (50–59, OR 6.012, 95%CI 1.410–25.629; 60–69, OR 13.915, 95%CI 3.381–57.267;≥70, OR 31.267, 95%CI 7.472–130.83), married(OR 1.780, 95%CI 1.186–2.672), LDL-C(OR 2.015, 95%CI 1.443–2.814), and HDL-C(OR 2.130, 95%CI 1.360–3.338). According to the coefficients generated from the multivariable logistic regression analysis, ROC curves were plotted. The area under the curve (AUC) was 0.741, as shown in Fig. 2.

**Table 3:** Determinants of Carotid Plaque Derived from Stepwise multivariable Logistic Regression Analysis

Variable	B	Odds Ratio(95% CI)	*P* Value	Score
Sex (male)	0.676	1.966 (1.406–2.749)	**<0.001**	1
age				
40–49	NA	1.0(Reference)	**<0.001**	0
50–59	1.794	6.012 (1.410–25.629)	**0.015**	3
60–69	2.633	13.915 (3.381–57.267)	**<0.001**	5
≥ 70	3.443	31.267 (7.472–130.83)	**<0.001**	6
marriage (married)	0.577	1.780 (1.186–2.672)	**0.005**	1
Diabetes mellitus	0.205	1.227 (0.785–1.920)	0.370	NA
Overweight or obesity	−0.248	0.780 (0.555–1.098)	0.154	NA
FPG (mmol/L)				
≤ 6.1	NA		0.547	NA
6.11~6.99	0.189	1.208 (0.751–1.945)	0.436	NA
≥ 7.0	0.253	1.287 (0.788–2.102)	0.313	NA
TC(> 5.2 mmol/L)	0.364	1.439 (0.933–2.221)	0.100	NA
LDL-C (>3.12 mmol/L)	0.701	2.015 (1.443–2.814)	**<0.001**	1
HDL-C (<1.04 mmol/L)	0.756	2.130 (1.360–3.338)	**0.001**	1

For these 5 risk factors, marriage (married) had the lowest regression coefficients 0.577, we scored it 1 point. Other 4 factors’ scores were calculated by dividing coefficients by 0.577 and then rounded to the nearest integers. Using this scoring system, we got a score for each subject from derivation set. Scores of all the subjects were used to plot ROC curve, and determine the prediction power of unstable carotid plaque and the best cutoff score by Youden index. The AUC was 0.738. The Yoden index of score was calculated by sensitivity plus specificity. The best predictive value of score was 6.5, sensitivity was 71.8%, and specificity was 63.0%, as shown in Fig. 2.

**Fig. 3:**
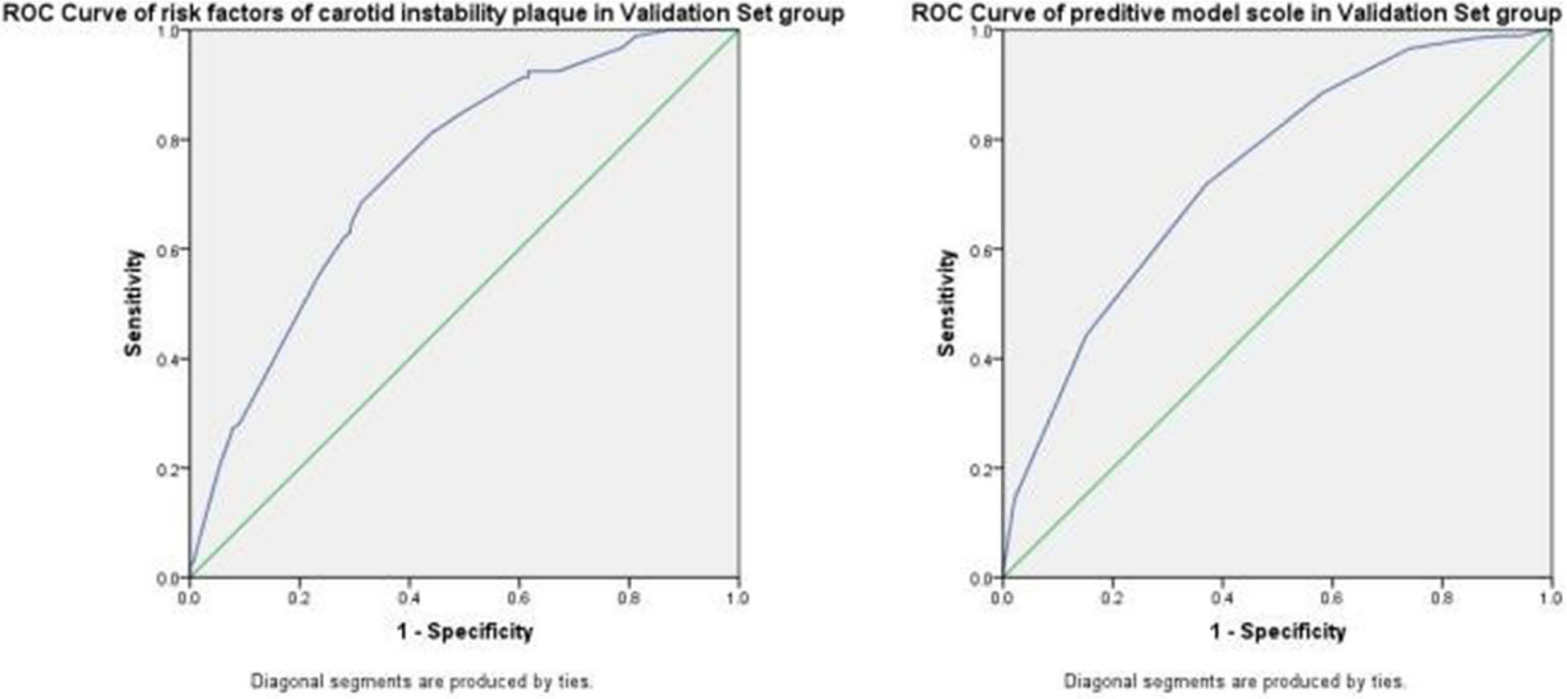
The ROC curve of Validation Set group for risk factors and predictive model Scoring in high risk group of strokes

The ability of the predictive model to discriminate between subjects with and without unstable carotid plaque was evaluated in a separate validation set comprising 947 persons (mean age = 60.4 ± 8.7 years), 92 (9.7%) of whom had unstable carotid plaques. The AUC was 0.743. According to the scoring system, we got a score for each subject from validation set. The scores of all the subjects were used to generate ROC curve. The AUC was 0.737. The Yoden index of score was calculated by sensitivity plus specificity. The best predictive value of score was still 6.5, sensitivity was 76.1%, and specificity was 63.6%, as shown in Fig. 3.

## Discussion

This study explored the risk factors of unstable carotid plaque in asymptomatic patients with high risk of stroke and developed a scoring system. This is the first time that a quantitative scale was developed to assess the risk of unstable carotid plaques. Previous studies focused on CIMT or carotid artery stenosis. Traditional risk factors such as hypertension, diabetes, smoking, etc., were often used in various prediction systems, however, serological indicators and personal history (education level, marriage) were rarely included. Patients with moderate to severe stenosis may benefit from carotid endarterectomy and carotid artery stenting, however they are relatively rare. So, if we pay more attention to unstable carotid plaques, we may identify more high-risk patients with lower level of stenosis who may benefit from aggressive primary interventions, such as healthy lifestyle, control of chronic diseases and risk-reducing medications [14].

In this study, marriage was associated with an increased prevalence of unstable carotid plaque both by univariate and multivariable analysis. It is the first time that marriage was associated with unstable carotid plaque. Yue W et al. investigated potential association between carotid artery stenosis and cognitive impairment among patients with acute ischemic stroke, including one subgroup analysis of the relationship between marriage and severity of carotid stenosis. The result showed that marriage was closely associated with severe stenosis of carotid artery, and the association was statistically significant (*P* = 0.0471) [15]. We noticed that this study was from Tianjin, China. This association may have some relationship to the Chinese tradition, as marriage can improve living quality, especially in terms of eating habits. Another possible reason is that subjects filled out questionnaires by themselves, which might not be very accurate.

Gender is another independent risk factor for unstable carotid plaque in individuals with high risk of stroke. Our study indicated male was a risk factor for unstable carotid plaque in individuals with high risk of stroke, which was similar to the outcome of a cohort from Taiwan [16]. On the other hand, a cohort from America indicated that men and women had equal risk for ACS [6]. Such discrepancies may be attributed to the racial difference in these studies, or the small sample size of our study.

Previous studies [17, 18] founded that older age, smoking, peripheral arterial disease, hypercholesterolemia, hypertension, Diabetes mellitus, and coronary artery disease were associated with occult carotid stenosis of > 50% or 60%. Our study found that diabetes mellitus was a risk factor for unstable carotid plaque by univariate analysis, however, our analysis showed that other traditional risk factors such as hypertension, HbA1c, homocysteine and smoking were not associated with unstable carotid plaque. The cause of stroke is complicated, carotid thromboembolism is only part of it (accounting for up to 20%), which is caused by carotid plaque or stenosis, especially unstable carotid plaque [19]. For that reason, the traditional risk factors may cause stroke, but may not necessarily cause unstable carotid plaque in this study.

The framingham heart study showed that the occurrence of atherosclerosis was negatively correlated with the level of HDL-C and positively correlated with the level of LDL-C [20]. This is similar to our research. Unstable plaque is a special type of atherosclerosis. LDL-C can damage endothelial cells and smooth muscle cells through oxidation, modification and glycosylation, start and maintain the inflammatory response of vascular wall, so as to develop atherosclerosis [21]. HDL-C can transport cholesterol from surrounding tissues (including macrophages and atherosclerotic plaques) to the liver for recycling or excretion in the form of cholic acid. This process is called reverse cholesterol transport (RCT). Through RCT, it can reduce the deposition of lipids in the blood vessel wall, thus reducing the cholesterol level in plasma and blood vessel wall and reducing the occurrence of atherosclerosis [22].

There are different viewpoints on the screen of ACS. Hill [23] reported that the prevalence of ACS is low, about 1–8%. With the higher degree of stenosis, the incidence of ACS is lower. Greco [6] reported that the prevalence of ACS (> 50% stenosis) is 2.4%. Given the low prevalence of carotid stenosis, many organizations do not recommend screening in the general population [3–5]. Meanwhile, other researchers [24, 25] suggested screening, especially for those with multiple risk factors for atherosclerosis, such as hypertension, hyperlipidemia, family history of atherosclerosis or ischemic stroke before 60 years of age and smoking. Jones [26] reported that the prevalence of carotid plaque was 21.0% in a total of 173 middle-aged subjects, which was similar to our findings. In this study, our subject’s average age was 60 years old, and the prevalence of carotid plaque and unstable carotid plaque was 35.2 and 9.4%, respectively. We believe that the occurrence of carotid plaque and stenosis is highly related to age. The older the age is, the higher the incidence is. This is also well reflected in our model. When the age is more than 70 years old, the value of the prediction model is as high as 7 points.

Our study developed a simple and handy scoring system. When individuals with high risk of stroke get a score of more than 6.5 points, carotid artery ultrasound screening should be recommended. Previous prediction models [6, 7] were only generated through medical history inquiry. In this study, objective blood indicators were included in the prediction model, which reduced subjective bias and made it more accurate.

However, certain inadequacies of this study cannot be ignored. First, although this study was carried out throughout 21 Communities in Nanjing, it’s still a single-center cross-sectional study, therefore cannot reflect the national prevalence and cannot tell the progression of unstable carotid plaque. Second, for the lack of time and staff, the sizes of the plaques were not measured in detail, and the plaque characteristics were not evaluated by 3D probe either. Third, some biomarkers are not fully included in this study, such as hs-CRP, which is a very important indicator of CV disease. Lastly, follow-up data of this population are being compiled, especially with regard to interventions (medication and surgery) and the occurrence of stroke. Regardless of these limitations, this study still provided solid evidence of the risk of unstable carotid plaque in individuals with high risk of stroke, and would be perfected in the future.

## Conclusions

The prevalence of carotid artery plaque as well as unstable plaque is high in population with high risk of stroke. It is necessary to screen carotid artery by Doppler ultrasonography in this population for early intervention. We developed a scoring system ranging from 0 to 10. When a resident’s score exceeds 6.5, the probability of having unstable carotid plaque is high, thus Doppler ultrasound examination of the carotid artery should be conducted as soon as possible and interventions should be introduced according to the guidelines.

## Supplementary information

**Additional file 1.** The questionnaire of CNSSPP. This is the questionnaire of CNSSPP. It includes the following contents: demographic information, preliminary screening information, and re-screening information. After preliminary screening, it was suggested that the high-risk population (Including previous stroke and TIA individuals) should be re-screened, otherwise the screening would be terminated.

**Additional file 2.** The results of gender age matched control group. This is the result of the study on the gender age matched control group (1:1), which can completely correct the influencing factors of gender and age. The result show that whether or not adjusted for age and gender, married, a higher level of LDL-C and a lower level of HLD-C were both the independent risk factors of carotid unstable plaque.

## Data Availability

The datasets used and/or analysed during the current study are available from the corresponding author on reasonable request.

## Abbreviations

ACS: Asymptomatic carotid stenosis
CNSSPP: China National Stroke Screening and prevention project
CVD: Cardiovascular diseases
FPG: Fasting plasma glucose
Hcy: Homocysteine
TC: Total cholesterol
HDL-C: High-density lipoprotein cholesterol
LDL-C: Low-density lipoprotein cholesterol
HbA1C: Hemoglobin A1C
TG: Triglyceride
ROC: Receiver operator characteristic
AUC: Area under the curve
