# Supplementary material for: A model to predict unstable carotid plaques in population with high risk of stroke
Source: BMC Cardiovasc Disord. 2020 Apr 7;20:164. doi: 10.1186/s12872-020-01450-z (PMC7137419; doi:10.1186/s12872-020-01450-z)
Supplement: Supplementary file 1 — Additional file 1. The questionnaire of CNSSPP. This is the questionnaire of CNSSPP. It includes the following contents: demographic information, preliminary screening information, and re-screening information. After preliminary screening, it was suggested that the high-risk population (Including previous stroke and TIA individuals) should be re-screened, otherwise the screening would be terminated. [file 12872_2020_1450_MOESM1_ESM.doc]

**2014 Risk assessment form of screening and intervention project for high risk population of stroke**

(For whole group screening in urban communities and rural townships over 40 years of age)

On-site survey paper version

Informed Consent: I have read the informed consent form and have volunteered to participate in this screening and intervention program, agreeing to use the data for the statistical, analytical and scientific work of the National Health Administration. Signature:

1. Archive information

| 1.1 Basic information | | | | | | | | | | | |
| --- | --- | --- | --- | --- | --- | --- | --- | --- | --- | --- | --- |
| Name of medical institution: | | | | | Date: . . . . . . . . . . . . . . . . . . . . . . . . . . . . . . . . . . . . . . . | | | | | Community: Urban , Rural | |
| Screener : | | | | | Qc : | | | | | | |
| 1. 2 Demographic information | | | | | | | | | | | |
| Name: | Gender: | | Ethnic: | | | | Idcard 12nununumnumber : . . . . . . . . . . . . . . . . . . . . . . . . . . . . . . . . . . . . . . . . . . . . . . . . . . . . . . . . . . . . . | | | | |
| Marital status | | | | Education: | | | | | | | |
| Occupation | | | | | | | | | | | |
| Average monthly personal medical expenses (excluding health insurance expenses):  Under 500 yuan , 500-1000 yuan , 1001-3000 yuan , 3001-5000 , 5001-10000 yuan , 10000 yuan or more Unknown | | | | | | | | | | | |
| The main medical payment methods: | | | | | | | | | | | |
| 1.3 Communications and contact information | | | | | | | | | | | |
| Address: | | | | | | | | Zip code : . . . . . . . . . . . . . . . . . . . . . . . . . . . . . | | | |
| Current residence address: | | | | | | | | Zip code : . . . . . . . . . . . . . . . . . . . . . . . . . . . . . | | | |
| Phone: . . . . . . . . . . . . . . . . . . . . . . . . . . . . . . . . . . . . . . . . . . . . . | | | | | | Mobilephone: . . . . . . . . . . . . . . . . . . . . . . . . . . . . . . . . . . . . . . | | | | | Email (optional): |
| Primary contact name: | | Relationship with me : parents , spouses and children , brothers and sisters , i , i , and i | | | | | | | Contact Phone: | | |

2. Initial screening information

| 1 | Hypertension (Blood pressure≥140/90 mmHg Or taking antihypertensive drugs): □YES □NO | | | | | | | |
| --- | --- | --- | --- | --- | --- | --- | --- | --- |
| 2 | Dyslipidemia (triglyceride ≥2.26 mmol/L, total cholesterol ≥6.22 mmol/L, high-density lipoprotein cholesterol <1.04 mmol/L, lowdensity lipoprotein cholesterol ≥4.14 mmol/L, self-reported diagnosis of dyslipidemia, or taking cholesterol-lowering medications) | | | | | | □NO □Unknown | |
| 3 | Diabetes: □YES □NO | | | | | | | |
| 4 | Atrial fibrillation: □YES □NO | | | | | | | |
| 5 | History of smoking: □YES □NO | | | | | | | |
| 6 | overweight or Obesity (BMI26kgs/m2): □YES □NO | | | | | | | |
| 7 | Lack of exercise or light manual labor (Exercise times < 3 times / week and < 30 minutes / time; participation in industrial and agricultural labor is regarded as exercise): □YES □NO | | | | | | | |
| 8 | Family History of Stroke□YES □NO | | | | | | | |
| Ⅰ. | Previous history of stroke: □YES □NO | | | | | | | |
| Ⅱ. | Previous history of transient ischemic attack □YES □NO | | | | | | | |
| screening preliminary results  (Systembuild) | | Risk classification | Stroke | TIA | n≥3 High-Risk | Media-risk | | Low-risk |
|  |  | Danger identification |  | | |  | |  |
|  |  | Management classification | "Fundmanagement | | | Standardized management | | Health Management |

3. Re-screening information

(People at high risk of stroke are rated by initial screening, and the relevant medical history is detailed based on the results of the initial screening, including previous stroke,TIA patients, and people with risk factors n≥3)

| 3.1Other important medical history | | | | |
| --- | --- | --- | --- | --- |
| Past stroke | | | □YES  type Stroke (optional): | □NO |
| transient ischemic attack | | | □YES  Number of TIA: | □NO |
| 3.1.3 | Hypertension | □YES | | □NO |
|  |  | Time of diagnosis: . . . . . . . . . . . . . . . . . . . . . . . . . . . . . . . . . . . . . . .  Take antihypertensive drugs in two weeks: □YES □NO | | |
| 3.1.3 | Dyslipidemia | □YES | | □NO |
|  |  | Time of diagnosis: . . . . . . . . . . . . . . . . . . . . . . . . . . . . . . . . . . . . . . .  Take lipid-lowering drugs in two weeks: □YES □NO | | |
| 3.1.3 | Diabetes | □YES | | □NO |
|  |  | Time of diagnosis: . . . . . . . . . . . . . . . . . . . . . . . . . . . . . . . . . . . . . . .  Take hypoglycemic drugs in two weeks: □YES □NO | | |
| 3.1.4 | Other heart diseases | □YES | | □NO |
|  |  | Heart disease type (optional):  Coronary heart disease , rheumatism heart disease (including combined valve lesions) , myocardial disease , atrial fibrillation , other types of arrhythmia , other types of heart disease | | |

| 3.2 Family History | | | |
| --- | --- | --- | --- |
| 3.2.1 | Stroke | □YES | □NO |
|  |  | Relationship with me: parents, children, siblings, other relatives | |
| 3.2.2 | Coronary heart disease | □YES | □NO |
|  |  | Relationship with me: parents, children, siblings, other relatives | |
| 3.2.3 | Hypertension | □YES | □NO |
|  |  | Relationship with me: parents, children, siblings, other relatives | |
| 3.2.4 | Diabetes | □YES | □NO |
|  |  | Relationship with me: parents, children, siblings, other relatives | |
| 3.2.5 | High blood lipids | □YES | □NO |
|  |  | Relationship with me: parents, children, siblings, other relatives | |
| Note: The above items, select sometimes, and my relationship is required, can be selected | | | |

| 3.3 Lifestyle | | | | |
| --- | --- | --- | --- | --- |
| 3.3.1 | Smoking | □YES | | □NO |
|  |  | If you are smoking, for ___ years ___months, how many cigarettes per day are now?  If you have quit smoking, how long? | | |
| 3.3.2 | Drinking | □YES | □NO | |
|  |  | Alcohol history : regular heavy drinking (high lying liquor50 degrees,3 times / week, 2 two / times) - Occasional drinking  Length of alcohol consumption: | | |
| 3.3.3 | Exercise habits | Regular exercise or heavy manual work (including industrial and agricultural workers) - Lack of exercise or light manual labor (number of exercises and 3 times/week and slt;30 minutes/times) | | |
| 3.3.4 | Dietary habits | Salty taste , taste oil eating vegetables ( s 5 days / weeks ,2 days / weeks) eating fruit (s) 3 days/week s little or occasionally to eat) Drink milk or yogurt (s.200ml/day and 5days/week s/week) | | |
| Take birth control pills (female) | | □YES □NO  Regular use of contraceptives : □often □occasionally | | |
| Take estrogen (female) | | □YES □NO  Take estrogen regularity : □often □occasionally | | |

4. Physical examination information

| Waist: ___ cm Hipline: ___cm Waist hip ratio _____ | |
| --- | --- |
| Height___ cm weight ___kg BMI_____ (kg/m^2^) | |
| Pulses ___ /min | |
| Blood pressure () | Left side SBP_____ mmHg DBP_____ mmHg  Right side SBP_____ mmHg DBP_____ mmHg |
| Heart hearing: | Heart Mursy : □YES □NO  Heart rhythm: neat Uneven |

5. Laboratory test information

| Check time: | | Inspection Agency :Base Hospital , Community or Township Hospital , Other | | |
| --- | --- | --- | --- | --- |
| Type | Check the indicator | Numerical | Unit | Identity |
| 5.1 Blood glucose | 6.1.1Fasting blood glucose | _______ | mmol/L | □↑/□normal /□↓ |
|  | 6.1.2 HbA1c | _______ | % | □↑/□normal /□↓ |
| 5.2 Blood Lipids | 6.2.1Triglyceride TG | _______ | mmol/L | □↑/□normal /□↓ |
|  | 6.2.2Total Cholesterol TCHO | _______ | mmol/L | □↑/□normal /□↓ |
|  | 6.2.3LDL-C | _______ | mmol/L | □↑/□normal /□↓ |
|  | 6.2.4HDL-C | _______ | mmol/L | □↑/□normal /□↓ |
| 5.3 Homocysteine HCY | | _______ | smol/L | □↑/□normal /□↓ |
|  | | | | |

6. Check the information

| 6.1 Electrocardiogram (heart hearing must do for people with arrhythmia) | | |
| --- | --- | --- |
| Check the results: | □Abnormal | □ normal |
|  | Exception type:  Atrial fibrillation , ischemic change , left ventricular hypertrophy , other types of arrhythmia , other diagnoses , please elaborate | |

| 6.2 Neck Vascular Ultrasound | | | | | | | | | | | | |
| --- | --- | --- | --- | --- | --- | --- | --- | --- | --- | --- | --- | --- |
| Check results : □all normal , □ there are abnormalities in any part | | | | | | | | | | | | |
| Exception type | | Abnormal items | Responsible lesions | | | | | | | | | |
|  |  |  | Left | | | | | Right | | | | |
|  |  |  | CCA | bulb | | | ICA | CCA | | bulb | ICA | |
| 7.2.1 | Inner membrane IMT | Thickening(IMT≥1.0mm) ( 0=no,1=yes) |  |  | | |  |  | |  |  | |
| 7.2.2 | plaque | Number | None (n-0) s single-shot(n-1) s/he multiple (n-2) | | | | | | | | | |
|  |  | Patterns (1=Irregular , 0=regular) |  | |  |  | |  |  | | |  |
|  |  | Ulcers (1= Yes , 0 =None) |  | |  |  | |  |  | | |  |
|  |  | Echo (1 =Strong Echo , 2= Medium Echo ,  3=Low Echo , 4 =Mixed Echo ) |  | |  |  | |  |  | | |  |
| 7.2.3 | Stenosis or occlusion | Stenosis rate (0=no stenosis; 1 =1-49%;2=50-69%; 3=70-99%; 4 closed) |  | |  |  | |  |  | | |  |
| 7.2.4 | Stent(CAS) | Postoperative (single):  Not done,0 to3 months, 3 to6 months, 6 to9 months, 9to 12 months, <2 years, <3 years, <5years |  | |  |  | |  |  | | |  |
| 7.2.5 | Stenosis after stent surgery | Stenosis rate(0=no stenosis; 1 =1-49%;2=50-69%; 3=70-99%; 4 closed) |  | | | | |  | | | | |
| 7.2.6 | Endothepha (CEA) | Postoperative (single):  Not done,0 to3 months, 3 to6 months, 6 to9 months, 9to 12 months, <2 years, <3 years, <5years |  | |  |  | |  |  | | |  |
| 7.2.7 | CEA postoperative stenosis | Stenosis rate(0=no stenosis; 1 =1-49%;2=50-69%; 3=70-99%; 4 closed) |  | | | | |  | | | | |

7. mRS score (improved Rankin scale, only stroke patients need to fill out)

| Assessed Time: | The no. times | Inspection Agency :Base Hospital , Community or Township Hospital , Other | |
| --- | --- | --- | --- |
| Options (Radio) | | | Score value |
| Completely asymptomatic | | | 0 |
| Despite symptoms, there are no obvious dysfunctions and can complete all daily work and life | | | 1 |
| Mild disability, not being able to complete all pre-disease activities, but without help taking care of your daily life | | | 2 |
| Moderate disability, need sit-in, but able to walk independently | | | 3 |
| Severely disabled, unable to walk independently, unable to meet the needs of their daily lives without the help of others | | | 4 |
| Severe disability, persistent bed, incontinence, require severance and attention, and rely entirely on others for daily life | | | 5 |
| mRS Score | | |  |
