# Supplementary material for: A model to predict unstable carotid plaques in population with high risk of stroke
Source: BMC Cardiovasc Disord. 2020 Apr 7;20:164. doi: 10.1186/s12872-020-01450-z (PMC7137419; doi:10.1186/s12872-020-01450-z)
Supplement: Supplementary file 2 — Additional file 2. The results of gender age matched control group. This is the result of the study on the gender age matched control group (1:1), which can completely correct the influencing factors of gender and age. The result show that whether or not adjusted for age and gender, married, a higher level of LDL-C and a lower level of HLD-C were both the independent risk factors of carotid unstable plaque. [file 12872_2020_1450_MOESM2_ESM.doc]

**The results of gender age matched control group**

We established a gender and age matched control group (1:1), which can completely correct the influencing factors of gender and age. We used conditional logistic regression to analyze the risk factors, as shown in below Table 1. The results show that these risk factors (married, diabetes mellitus, a higher level of FPG /TC/ LDL-C, a lower level of HLD-C) were similar to before. Hypercholesterolemia may the risk factors of Carotid Vulnerable Plaque (p=0.076). Overweight or obesity would no longer be a risk factor (p=0.155). This may because the small sample size of matching group (1:1).

**Table 1 Comparison of Demographic and Risk Factor Profiles in Participants with and Without Carotid Instability Plaque in the Derivation Set (gender and age matched control group 1:1 )**

| Variable | With Carotid Vulnerable Plaque  (n=174) | Without Carotid Vulnerable Plaque(control)  (n=174) | P Value | OR（95%CI） |
| --- | --- | --- | --- | --- |
| Sex (male) | 109 (62.6%) | 106 (60.9%) | 0.825 | 1.05 (0.681-1.619) |
| Mean age (years) | 65.9±8.0 | 65.9±7.4 | 0.975 | 1.00 (0.973-1.027) |
| Education level  (Primary school or below) | 67 (38.5%) | 66 (37.9%) | 0.910 | 1.026 (0.660-1.594) |
| Marriage(married) | 141(81.0%) | 122 (70.1%) | **0.018** | **1.864（1.110-3.128）** |
| Atrial fibrillation | 13 (7.5%) | 13 (7.5%) | 1.0 | 1.0（0.449-2.226） |
| Diabetes mellitus | 54 (31.0%) | 41 (23.6%) | **0.099** | **1.542（0.922-2.577）** |
| Hypertension | 145 (83.3%) | 147 (84.5%) | 0.763 | 0.913（0.505-1.650） |
| Hypercholesterolemia | 67 (38.5%) | 51 (29.3%) | **0.076** | **1.500（0.959-2.346** |
| Overweight or obesity | 61 (35.1%) | 75 (43.1%) | 0.155 | 0.745（0.497-1.117） |
| Smoking | 68 (39.1%) | 75 (43.1%) | 0.468 | 0.860（0.572-1.293） |
| Lack of Physical activity | 134 (77.0%) | 134 (77%) | 1.00 | 1.00（0.608-1.645） |
| Family history of strok | 54 (31.0%) | 45 (25.9%) | 0.300 | 1.273（0.807-2.008） |
| FPG(mmol/L) |  | | | |
| ≤6.1 | 101 (58.0%) | 120 (69.0%) | **0.081** | 1.0（Reference） |
| 6.11~6.99 | 29 (16.7%) | 22 (12.6%) | **0.118** | 1.656（0.880-3.118） |
| ≥7.0 | 44 (25.3%) | 32 (18.4%) | **0.060** | 1.715（0.979-3.004） |
| HbA1c (>6.5%) | 14 (8.0%) | 13 (7.5%) | 0.840 | 1.085（0.491-2.396） |
| Hcy(>15mmol/L) | 30 (17.2%) | 36 (20.7%) | 0.319 | 0.750（0.426-1.321） |
| TC(>5.2mmol/L) | 91 (52.3%) | 59 (33.9%) | **0.001** | **2.143（1.368-3.356）** |
| LDL-C(>3.12mmol/L) | 97 (55.7%) | 59 (33.9%) | **<0.001** | **2.407（1.537-3.771）** |
| HDL-C(<1.04mmol/L) | 32 (18.4%) | 12 (6.9%) | **0.003** | **2.818（1.417-5.607）** |
| TG(>1.7mmol/L) | 59 (33.9%) | 46 (26.4%) | 0.145 | 1.394（0.891-2.180） |

Additional, those seven risk factors (married, diabetes mellitus, hypercholesterolemia, a higher level of FPG/TC/ LDL-C, a lower level of HLD-C) were included in the multivariable logistic regression model (Stepwise forward), as shown in below Table 2. The results show that these risk factors (Married, a higher level of LDL-C, a lower level of HLD-C) were statistical significance with carotid unstable plaque, which is also similar to before.

Table 2. Determinants of Carotid Plaque Derived from Stepwise multivariable Logistic Regression Analysis

| Variable | B | Odds Ratio(95% CI） | P Value |
| --- | --- | --- | --- |
| marriage(married) | 0.518 | 1.678（1.125-2.503） | **0.011** |
| Diabetes mellitus | 0.179 | 1.196 (0.784-1.824) | 0.407 |
| hypercholestero | -0.117 | 0.889(0.641-1.233) | 0.481 |
| FPG(mmol/L) |  |  |  |
| ≤6.1 | NA |  | 0.279 |
| 6.11~6.99 | 0.246 | 1.279 (0.808-2.025) | 0.294 |
| ≥7.0 | 0.357 | 1.429 (0.897-2.278) | 0.133 |
| TC(>5.2mmol/L) | 0.265 | 1.303 (0.865-1.963) | 0.205 |
| LDL-C(>3.12mmol/L) | 0.373 | 1.453（1.016-2.078） | **0.041** |
| HDL-C(<1.04mmol/L) | 0.662 | 1.862（1.205-2.878） | **0.005** |

Therefore, whether or not adjusted for age and gender, married, a higher level of LDL-C and a lower level of HLD-C were both the independent risk factors of carotid unstable plaque. Those results would support the analyses and the conclusions of this study. Atherosclerosis is related to age and gender, especially age. If a predictive scoring system does have the age and gender, it will be an imperfect model. In this study, age, gender, marriage, LDL and HDL were retained as the indicators of the final prediction model.
